# The quandary of diagnosing mathematical difficulties in a generally low performing population

**DOI:** 10.1590/1980-57642021dn15-020015

**Published:** 2021

**Authors:** Mariuche Rodrigues de Almeida Gomides, Isabella Starling-Alves, Giulia Moreira Paiva, Leidiane da Silva Caldeira, Ana Luíza Pedrosa Neves Aichinger, Maria Raquel Santos Carvalho, Julia Bahnmueller, Korbinian Moeller, Júlia Beatriz Lopes-Silva, Vitor Geraldi Haase

**Affiliations:** 1 Graduate Program in Psychology: Cognition and Behavior, Universidade Federal de Minas Gerais - Belo Horizonte, MG, Brazil.; 2 Laboratory of Developmental Neuropsychology, Universidade Federal de Minas Gerais - Belo Horizonte, MG, Brazil.; ³ Educational Psychology Department, University of Wisconsin-Madison - Madison, United States.; 4 Graduate Program in Neuroscience, Universidade Federal de Minas Gerais - Belo Horizonte, MG, Brazil.; 5 Graduate Program in Genetics, Universidade Federal de Minas Gerais - Belo Horizonte, MG, Brazil.; 6 Department of Genetics, Ecology and Evolution, Universidade Federal de Minas Gerais, Belo Horizonte, MG, Brazil.; 7 Centre for Mathematical Cognition, School of Science, Loughborough University - Loughborough, United Kingdom.; 8 Leibniz-Institut für Wissensmedien - Tübingen, Germany.; 9 LEAD Graduate School & Research Network, University of Tübingen - Tübingen, Germany.; 10 Department of Psychology, University of Tübingen - Tübingen, Germany.; 11 Instituto Nacional de Ciência e Tecnologia sobre Comportamento, Cognição e Ensino - São Carlos, SP, Brazil.

**Keywords:** diagnosis, dyscalculia, learning disabilities, mathematics, diagnóstico, dificuldades de aprendizagem, discalculia, matemática

## Abstract

Brazilian students’ mathematical achievement was repeatedly observed to fall below average levels of mathematical attainment in international studies such as PISA.

**Objective:**

In this article, we argue that this general low level of mathematical attainment may interfere with the diagnosis of developmental dyscalculia when a psychometric criterion is used establishing an arbitrary cut-off (e.g., performance<percentile 10) may result in misleading diagnoses.

**Methods:**

Therefore, the present study evaluated the performance of 706 Brazilian school children from 3^rd^ to 5^th^ grades on basic arithmetic operations addition, subtraction, and multiplication.

**Results:**

In line with PISA results, children presented difficulties in all arithmetic operations investigated. Even after five years of formal schooling, less than half of 5^th^ graders performed perfectly on simple addition, subtraction, or multiplication problems.

**Conclusions:**

As such, these data substantiate the argument that the sole use of a psychometric criterion might not be sensible to diagnose dyscalculia in the context of a generally low performing population, such as Brazilian children of our sample. When the majority of children perform poorly on the task at hand, it is hard to distinguish atypical from typical numerical development. As such, other diagnostic approaches, such as Response to Intervention, might be more suitable in such a context.

## RESUMO.

O desempenho em matemática dos estudantes brasileiros mostra-se consistentemente abaixo da média mundial em estudos internacionais como o PISA.

**Objetivo:**

No presente artigo, argumenta-se que um baixo desempenho geral na matemática, a exemplo dos estudantes brasileiros, pode interferir no diagnóstico de discalculia do desenvolvimento quando um critério puramente psicométrico é usado para estabelecer um ponto de corte arbitrário (por exemplo, desempenho<percentil 10), o que pode resultar em falsos diagnósticos.

**Métodos:**

Para tanto, investigou-se o desempenho de 706 estudantes brasileiros do 3º ao 5º ano escolar em operações aritméticas básicas de adição, subtração e multiplicação.

**Resultados:**

De forma consistente com os resultados do PISA, as crianças apresentaram dificuldades em todas as operações aritméticas investigadas. Mesmo após cinco anos de escolarização formal, menos da metade dos estudantes do 5º ano foi capaz de completar a tarefa envolvendo cálculos simples de adição, subtração ou multiplicação.

**Conclusões:**

Dessa forma, os resultados reforçam o argumento de que o uso exclusivo de um critério psicométrico pode não ser apropriado para o diagnóstico de discalculia no contexto de uma população com desempenho geral baixo, como no caso crianças brasileiras da presente amostra. Quando a maioria das crianças tem um desempenho aquém do esperado, torna-se difícil distinguir o desenvolvimento numérico atípico do típico. Portanto, outras abordagens diagnósticas, como Resposta à Intervenção, podem ser mais adequadas em tal contexto.

## INTRODUCTION

Mathematics is an important predictor of scientific and technological development, which is important for success in competitive global economies.^1^ For this reason, many countries have increased investments in basic mathematical education.^1^ ^,^ ^2^ ^,^ ^3^ Despite increased international recognition and higher investments in mathematical education, mathematical achievement in several countries remains a cause for concern.^4^ This is especially the case in Brazil.^5^ According to Programme for International Student Assessment (PISA) scores, no significant improvement has been observed in mathematics achievement of Brazilian students from 2003 to 2018. Results of PISA 2018 indicated that performance of Brazilian students in mathematics was significantly below Organisation for Economic Co-operation and Development (OECD) average.^6^ Moreover, the majority of students assessed scored below level 2 of math proficiency, which is considered the minimum necessary for young people to fully exercise their citizenship.^6^ Finally, PISA results also showed another alarming result: the upper half of Brazilian students (i.e., performing above percentile 50) still performed worse than the lower half of students (i.e., performing below percentile 50) from countries scoring highest in PISA 2018 such as South Korea, Finland, and Canada.^7^

A cornerstone for developing more advanced mathematical abilities is the mastery of the basic arithmetic operations: addition, subtraction, and multiplication.^8^ ^,^ ^9^ When children start learning basic arithmetic operations, they usually use rather effortful and error-prone procedural strategies, mostly based on (finger) counting.^10^ With practice, children become able to use more sophisticated procedural strategies (e.g., based on mental calculation and using composition/decomposition of numbers, for example “16+7=16+4=20+3=23”) and may even retrieve solutions from long-term memory for specific problems (e.g., tie problems such as “4+4”) or operations such as multiplication. However, some children persistently struggle to learn arithmetic.

Difficulties in learning basic arithmetic operations have been associated with dyscalculia, which reflects a circumscribed disability in handling numbers and arithmetic operations.^11^ A substantial number of school-aged children (i.e., between 3 and 6%, depending on the study)^12^ suffer from this learning disability, characterized by severe and persistent difficulties in mathematical learning that cannot be explained by primary causes such as intellectual deficits, emotional/motivational problems, and/or lack of adequate schooling.^11^ Dyscalculia is characterized by difficulties with the most basic aspects of mathematics, such as the ability to understand and discriminate quantities,^13^ ^,^ ^14^ ^,^ ^15^ ^,^ ^16^ read and write numbers.^17^ ^,^ ^18^ Additionally, difficulties with acquiring arithmetic facts knowledge are a cardinal symptom of dyscalculia.^19^ ^,^ ^20^

So far, there are no biological or cognitive markers sufficiently reliable to diagnose dyscalculia. Therefore, standardized tests of mathematical achievement are the most popular tool for diagnosing dyscalculia.^21^ According to the Diagnostic and Statistical Manual of Mental Disorders (DSM-5),^11^ dyscalculia can be diagnosed when: (a) performance in standardized tests of mathematical achievement falls below a specific cut-off point (i.e., psychometric criterion), (b) mathematical difficulties compromise the psychosocial adaptation of the individual (i.e., psychosocial impairment criterion), and (c) mathematical difficulties cannot be attributed to other primary causes as mentioned above (i.e., clinical exclusion criterion). Importantly, the clinical exclusion and the psychosocial impairment criteria have the downside of being subjective, and thus may well depend on the clinician’s experience. However, the psychometric criterion is not less problematic.

The psychometric approach has important limitations.^22^ ^,^ ^23^ ^,^ ^24^ So far, different cut-offs in standardized mathematical tests, ranging from the 5^th^ to the 35^th^ percentiles, have been employed across different studies (see^25^ for a review). Furthermore, the use of standardized mathematical achievement tests, alone, does not provide information about potentially impaired neurocognitive processes underlying dyscalculia.^26^ Instead, such tests usually only allow for the classification of a child’s achievement as viewed against a comparison group (e.g., children of the same age or school grade).

Given the overall low mathematics achievement consistently observed among Brazilian children,^6^ using a psychometric approach may lead to false-positive diagnoses of dyscalculia as performance below a specific percentile may not allow to differentiate between atypical and typical poor performance. As such, the main purpose of the present study was to assess the performance of Brazilian primary school children on basic arithmetic operations and evaluate how this information can be used to diagnose dyscalculia in the Brazilian context. Therefore, we assessed performance of 3^rd^, 4^th^, and 5^th^ graders on basic arithmetic operations, including addition, subtraction, and multiplication, to evaluate the acquisition of these abilities across grades. With this approach, we aimed at finding out by which grade children achieve proficiency in basic arithmetic operations. In the following, we first present detailed information on the study before reporting and comparing results operations and grades. Finally, we discuss the challenge of diagnosing dyscalculia in Brazil, using the psychometric criterion, considering the present results.

## METHODS

### Participants

Participants were 706 children with typical general cognitive abilities (above percentile 15 in CPM-Raven)^27^ attending third to fifth grade (Mean_age in years_=9.11, ±1.01; 55.5% girls), selected from 13 public schools and one private school in Belo Horizonte, Minas Gerais, the state with the third highest income in Brazil.^28^ All participants gave oral assent prior to testing and provided informed consent signed by their parents or primary caregivers. The study was approved by the local Research Ethics Committee.

### Task and procedure

This study was part of a more comprehensive project investigating the development of mathematical abilities of school-age children in Brazil. In this project, children completed a battery of tasks measuring general cognitive abilities (e.g., executive functions), and numerical and mathematical abilities (e.g., nonsymbolic and symbolic magnitude processing and numerical transcoding). For the purpose of this article, we specifically focused on the results of the Basic Arithmetic Operations Task (BAOT), which was assessed individually in a quiet separate room at participants’ school.

The BAOT consisted of 27 addition, 27 subtraction, and 28 multiplication problems. Problems of each operation were presented in fixed order of increasing difficulty on separate sheets of paper. Children were instructed to solve as many problems as possible within a 2-minute time limit per operation. The percentage of correctly solved items (i.e., the number of correctly solved problems divided by the total number of problems in the task) for each operation type was used as the dependent variable (for more information, see^29^).

The time limit in BAOT was established based on the performance of 16 college students (Mean_*age in years*_ =22.93, ±2.56, 62.5% female), who mastered basic operations. Results showed that adults were well able to solve all addition, subtraction, and multiplication problems within 2 minutes (i.e., addition: Mean_seconds_=59, ±9.83; subtraction: Mean_seconds_=73, ±13.28; multiplication: Mean_seconds_=83, ±13.60), with hardly any errors (i.e., percentage of correctly solved items for addition: Mean_corrects_=0.99, ±0.01; subtraction: Mean_corrects_=0.97, ±0.05; multiplication: Mean_corrects_=0.92, ±0.08). Based on these estimates, we expected that children fairly fluent in solving basic arithmetic operations should be able to complete all problems within the 2-minute time limit per operation type.

## RESULTS

In our analysis, we evaluated performance of 3^rd^, 4^th^ and 5^th^ graders in the BAOT operation types. First, we present descriptive analyses for each operation before the results of a mixed-model repeated measure ANOVA aiming to discern the influences of the independent between-participants variable grade level (i.e., 3^rd^ *vs*. 4^th^ *vs*. 5^th^ grade) and the within-participants variable operation (i.e., addition vs. subtraction vs. multiplication) on the percentage of correctly solved items.

### Addition

**Figure 1.**
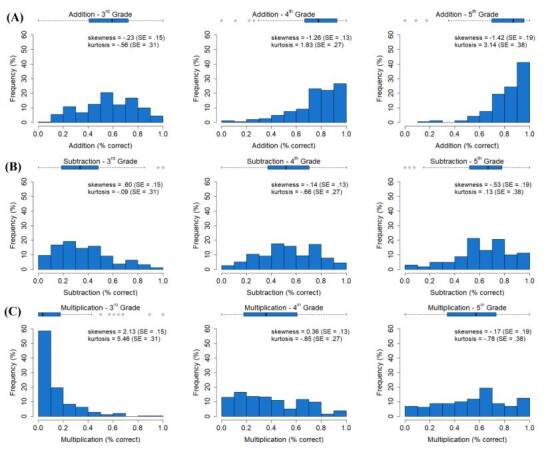
Children’s performance on addition, subtraction, and multiplication operations across grades.

In 3^rd^ grade, children were still learning basic addition, such that different scores were observed with similar frequencies in the task. In 4^th^ grade, children started to master addition, with higher scores being observed more frequently than lower scores. Similarly, in 5^th^ grade, higher scores were observed more frequently than lower scores, but children still did not reach perfect accuracy (Figure 1A). Tests of normal distribution (i.e., Kolmogorov-Smirnov, henceforth KS) indicated a non-normal distribution of addition scores for all three grades (KS_3rd grade_=1.51, p=0.02; KS_4th grade_=2.08, p<0.001; KS_5th grade_=2.01, p<0.01). These results suggest that performance on addition problems seemed to improve from 3^rd^ to 5^th^ grade. However, less than 50% of children in 5^th^ grade correctly solved more than 80% of the BAOT addition problems.

### Subtraction

Third graders presented difficulties with subtraction operations, such that the most frequent percentage of correct responses was below 50% (Figure 1B). In 4^th^ grade, a transition (i.e., similar frequencies for different scores) was observed, indicating that children were still learning subtraction. In 5^th^ grade, children started to improve their performance in subtraction, with scores above 50% of correct responses becoming more frequent. However, most children still achieved less than 75% of correct responses. KS tests indicated non-normal distributions of subtraction scores in the 3^rd^ (KS=1.47, p=0.03) and 4^th^ (KS=1.38, p=0.04) grades, but not in the 5^th^ grade (KS=1.12, p=0.16). Thus, similar to addition, results suggested an improvement in subtraction performance from 3^rd^ to 5^th^ grade. However, by 5^th^ grade, less than 20% of students were able to solve all items correctly, even though these only involved minuends up to 20.

### Multiplication

A floor effect was observed in 3^rd^ grade for multiplication, with most children not being able to solve any of the problems correctly (Figure 1C). In 4^th^ and 5^th^ grades, children started to learn multiplication operations, such that different scores were observed with similar frequencies in the task, suggesting only limited improvement between these grades. KS tests revealed a non-normal distribution of multiplication scores for 3^rd^ (KS=3.64, p<0.001) and 4^th^ (KS=1.62, p<0.01) graders and a distribution closer to normal for 5^th^ graders (KS=1.12, p=0.16). These results suggest that, despite some improvement in multiplication skills from 3^rd^ to 5^th^ grade, 5^th^ graders still do not master multiplication tables for single-digit numbers, with less than 20% of children with a maximum score in multiplication (Figure 1).

We considered the interval of 80 to 100% of correct responses as a criterion for fluency on BAOT operations. Then, we evaluated the percentage of children who met this criterion. Although this criterion was chosen more or less arbitrarily, we expected adults (i.e., as described above in the method section) and 5^th^ graders to be able to fluently solve the BAOT operations based on the results of previous studies (cf.^30^ ^,^ ^31^ ^,^ ^32^ ^,^ ^33^). By choosing the interval of 80 to 100%, we do, however, leave room for occasional careless mistakes, or situational or motivational digressions.

The majority of 3^rd^ graders (85.4%) did not master single-digit addition operations. This percentage drops considerably by 5^th^ grade, in which only 34% of children had not yet mastered basic addition operations. A smaller improvement was observed for 5^th^ graders with respect to subtraction and multiplication, in comparison to addition. For subtraction operations, 95.4% of 3^rd^ graders failed to meet our criterion, dropping to 78.5% in 5^th^ grade. For multiplication, 99.2% of 3^rd^ graders failed to meet the criterion, dropping to 80.6% in 5^th^ grade (Table 1).

**Table 1.** Percentage of children scoring above 80% of correct responses on addition, subtraction, and multiplication operations at each grade.

	Addition			Subtraction			Multiplication		
	3^rd^	4^th^	5^th^	3^rd^	4^th^	5^th^	3^rd^	4^th^	5^th^
80-90%	10.0	22.1	24.5	3.4	7.9	10.1	0.4	1.6	6.9
91-100%	4.6	26.6	41.5	1.2	4.6	11.4	0.4	3.9	12.5
80-100%	14.6	48.7	66.0	4.6	12.5	21.5	0.8	5.5	19.4

**Figure 2.**
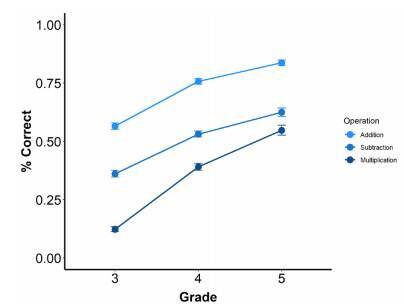
Mean percentage of correct responses for grades and operations. Error bars indicate standard erros.

Finally, the mixed-model ANOVA indicated a significant main effect of grade, F_(2, 703)_=154.4, p<0.001, ⴄ_p_^2^=0.17, with performance improving across grades. Pairwise comparisons indicated that 5^th^ graders’ scores were higher than those of 4^th^ and 3^rd^ graders, and 4^th^ graders’ scores were higher than those of 3^rd^ graders. There also was a significant main effect of operation, F_(2, 1321)_=1085.34, p<0.001, ⴄ_p_^2^=0.26. Pairwise comparisons indicated that addition operations were solved better than subtraction and multiplication operations and that subtraction operations were solved better than multiplication operations. Means and standard deviations are shown in Figure 2.

The interaction of grades and operation types was also significant, F_(4, 1321)_=20.36, p<0.01, ⴄ_p_^2^=0.01. To evaluate where this interaction of two three-levelled factors originated from, we followed the procedure suggested by Kirk,^34^ evaluating influences of grade level (i.e., 3^rd^ *vs*. 4^th^ *vs*. 5^th^ grade) on differences between arithmetic operations using univariate ANOVAs. The first ANOVA indicated that performance differences between addition and subtraction was not significantly influenced by grade, F_(2, 703)_=1.07, p=0.34, ⴄ_p_^2^=0.01. Importantly, results were different for performance differences between addition and multiplication, F_(2, 703)_=23.07, p<0.001, ⴄ_p_^2^=0.06, as well as subtraction and multiplication, F_(2, 703)_=31.61, p<0.001, ⴄ_p_^2^=0.08, for which the ANOVAs indicated significant effects of grade. Pairwise comparisons indicated that, for both addition and subtraction, differences with multiplication decreased as grade increased, with all pairwise comparisons being significant (p<0.05). In summary, this means that the significant interaction between grade and arithmetic operation reflects a decrease in performance differences between addition and multiplication, as well as between subtraction and multiplication, as grade increases whereas differences between performance in addition and subtraction did not change significantly across grades (Figure 2).

## DISCUSSION

In this study, we evaluated the performance of Brazilian children on basic arithmetic operations. Moreover, considering evidence showing that Brazilian students perform poorly in mathematics more generally, we aimed at evaluating the feasibility of diagnosing dyscalculia using the psychometric criterion. Our results indicated that a considerable percentage of primary school children did not master basic arithmetic operations by the end of fifth grade - even in a rather wealthy Brazilian region.^28^

As such, our findings are in line with the performance of Brazilian students on PISA, which repeatedly revealed average mathematical achievement to be below basic proficiency levels. However, rather than assessing specific mathematical abilities taught in school, the abilities measured by PISA are more generic and related to the use of mathematics in everyday life.^35^ Given that our participants were not able to solve basic arithmetic operations flawlessly, it may be the case that applying this kind of arithmetic knowledge to everyday situations, such as required by PISA, is challenging for Brazilian students.

The difficulties observed with the basic arithmetic operations in the present sample also have implications for the diagnosis of dyscalculia using a psychometric criterion. When the psychometric criterion is used, a more conservative percentile cut-off (e.g., ≤percentile 10) might allow the identification of children with severe and persistent mathematical difficulties.^25^ On the other hand, a more liberal criterion (e.g., ≤percentile 25) increases the chances of identifying children with less severe and persistent difficulties that are more likely associated with other causes.^25^

In the Brazilian context, with most children performing poorly on basic arithmetic operations, the psychometric criterion might become inappropriate for the diagnosis of dyscalculia. In these circumstances, the psychometric criterion can lead to both false-negative and false-positive diagnoses. False-negatives occur when children who have inherent difficulties are not distinguished from those classified as typical achievers. In contrast, false-positive occur when children whose difficulties are caused by factors such as poor education are diagnosed as having dyscalculia. Reducing both false-negatives and false-positives is important for providing services for children with more severe and persistent mathematical difficulties. Furthermore, under budget constraints, children formally diagnosed with developmental disorders are prioritized to participate in intervention programs.^36^

An alternative approach to the diagnosis of dyscalculia, increasingly adopted worldwide, is to base decisions not only on test scores but also consider children’s response to intervention (RTI).^4^ The RTI approach aims at identifying children at risk for mathematical learning difficulties as early as Kindergarten, to provide them with additional mathematical instruction in successive tiers of increasing intensity. This approach is both preventive and therapeutic. In this context, the diagnosis of dyscalculia is restricted to those children who do not respond to even the best and most intensive pedagogical efforts. RTI has the advantage of constraining the problem of learning difficulties to the school. However, its logistics are complex, expensive, and require personnel training and compliance from both teachers and children. Additionally, RTI has the drawback of potentially delaying recognition of serious health conditions possibly underlying mathematics learning difficulties (e.g., genetic syndromes), as children are usually referred to specialized services for their learning difficulties.

The low performance of our participants on basic arithmetic operations may be a result of external factors, such as socioeconomic status (SES)^37^ ^,^ ^38^ and educational experiences.^39^ However, specific evaluation of these was beyond the scope of this study. Nevertheless, SES was found to have a significant influence on a Brazilian national measure of mathematics achievement^40^ such that children with a better SES background outperformed those with lower SES. In line with this, we also observed a significant, but small, correlation of children's performance in addition (r=0.10, p<0.01) and multiplication (r=0.09, p<0.05) with SES in our sample. This corroborates the interpretation that poor performance observed for basic arithmetic operations in the present study may not only indicate MLD but also reflect influences of external educational factors. In this sense, effects of SES are also reflected in performance gaps observed between public and private schools, with private schools achieving scores higher than public schools and higher than the national average.^41^ ^,^ ^42^ Importantly, however, it should be noted that in addition to SES the gap between public and private schools may also be a result of different educational practices and school quality. Even though the use of a core curriculum is highly encouraged in Brazil,^43^ private schools usually push students harder and provide them with better educational and emotional support.

Educational experiences may also influence the performance in arithmetic operations. The Brazilian Ministry of Education (*Ministério da Educação* ENT#091;MECENT#093;) recently suggested a core curriculum, the National Common Core (*Base Nacional Comum Curricular* ENT#091;BNCCENT#093;), aiming to unify pedagogical principles and goals across the country.^43^ According to the BNCC, basic arithmetic operations are gradually introduced with increasing grade level, starting with addition in 1^st^ grade, subtraction in 2^nd^ grade, and multiplication in 3^rd^ grade. Formal strategies and procedures are recommended to be explicitly and systematically taught from 3^rd^ grade. It is expected that 4^th^ graders should be able to fluently implement formal algorithms in addition and subtraction. Conceptual aspects of arithmetic operations are explicitly and systematically taught only in 4^th^ grade. Remarkably, BNCC emphasizes the learning of conceptual and procedural arithmetic knowledge, whereas less effort is dedicated to promoting automatization of arithmetic facts. Despite the importance of conceptual and procedural knowledge, direct retrieval-based solutions were argued to be more efficient than calculation.^44^ Moreover, poor automatization of basic arithmetic operations has also been associated with difficulty in acquiring more complex mathematical abilities.^45^ ^,^ ^46^ This evidence highlights the importance of pedagogical practices, such as repetitive exercises with feedback and cumulative review, that promote automatization of arithmetic operations.^47^ As we used a speeded assessment, our results may be interpreted as reflecting difficulties with fluency or automatization, probably due to the lower emphasis on this in the Brazilian curriculum.

In this study, we evaluated the performance of Brazilian children on the basic arithmetic operations of addition, subtraction, and multiplication. Overall, most children presented difficulties in all arithmetic operations assessed. Children presented better scores in addition, compared to subtraction and multiplication, and 3^rd^ and 4^th^ graders were outperformed by 5^th^ graders in all three operations. However, 5^th^ graders still have not mastered these basic arithmetic operations fluently, with less than 50% of 5^th^ graders performing at 80% or above on addition, subtraction, or multiplication. This alarming scenario discourages the sole use of a psychometric criterion to diagnose dyscalculia. When the majority of children are performing poorly on a task, it is hard to differentiate those with dyscalculia from those whose poor performance is due to external factors, such as inadequate schooling. We question the use of the psychometric criterion as the only index of a developmental disability. Instead, RTI approaches might be better suited to the Brazilian context. In addition to contributing to clinical practice, these results might also inform educators and policy makers.
